# Sex differences in treatment strategy and adverse outcomes among patients 75 and older with atrial fibrillation in the MarketScan database

**DOI:** 10.1186/s12872-021-02419-2

**Published:** 2021-12-16

**Authors:** Vinita Subramanya, J’Neka S. Claxton, Pamela L. Lutsey, Richard F. MacLehose, Lin Y. Chen, Alanna M. Chamberlain, Faye L. Norby, Alvaro Alonso

**Affiliations:** 1 Department of Epidemiology, Rollins School of Public Health, Emory University, Atlanta, GA 30322 USA; 2 Division of Epidemiology and Community Health, School of Public Health, University of Minnesota, Minneapolis, MN USA; 3 Cardiovascular Division, Department of Medicine, University of Minnesota Medical School, Minneapolis, MN USA; 4 Department of Quantitative Health Sciences, Mayo Clinic, Rochester, MN USA; 5 Department of Cardiovascular Medicine, Mayo Clinic, Rochester, MN USA; 6 Department of Cardiology, Smidt Heart Institute, Cedars-Sinai Health System, Los Angeles, CA USA

**Keywords:** Atrial fibrillation, Anticoagulation, Rate control, Rhythm control, Sex differences, Heat failure, Stroke, Major bleeding

## Abstract

**Background:**

Women with atrial fibrillation (AF) experience greater symptomatology, worse quality of life, and have a higher risk of stroke as compared to men, but are less likely to receive rhythm control treatment. Whether these differences exist in elderly patients with AF, and whether sex modifies the effectiveness of rhythm versus rate control therapy has not been assessed.

**Methods:**

We studied 135,850 men and 139,767 women aged ≥ 75 years diagnosed with AF in the MarketScan Medicare database between 2007 and 2015. Anticoagulant use was defined as use of warfarin or a direct oral anticoagulant. Rate control was defined as use of rate control medication or atrioventricular node ablation. Rhythm control was defined by use of anti-arrhythmic medication, catheter ablation or cardioversion. We used multivariable Poisson and Cox regression models to estimate the association of sex with treatment strategy and to determine whether the association of treatment strategy with adverse outcomes (bleeding, heart failure and stroke) differed by sex.

**Results:**

At the time of AF, women were on average (SD) 83.8 (5.6) years old and men 82.5 (5.2) years, respectively. Compared to men, women were less likely to receive an anticoagulant or rhythm control treatment. Rhythm control (vs. rate) was associated with a greater risk for heart failure with a significantly stronger association in women (HR women = 1.41, 95% CI 1.34–1.49; HR men = 1.21, 95% CI 1.15–1.28, *p* < 0.0001 for interaction). No sex differences were observed for the association of treatment strategy with the risk of bleeding or stroke.

**Conclusion:**

Sex differences exist in the treatment of AF among patients aged 75 years and older. Women are less likely to receive an anticoagulant and rhythm control treatment. Women were also at a greater risk of experiencing heart failure as compared to men, when treated with rhythm control strategies for AF. Efforts are needed to enhance use AF therapies among women. Future studies will need to delve into the mechanisms underlying these differences.

**Supplementary Information:**

The online version contains supplementary material available at 10.1186/s12872-021-02419-2.

## Background

Atrial fibrillation (AF) is a common arrhythmia prevalent in adults over 65 years of age, with a twofold increase in prevalence every decade of life beyond 50 years [1]. Sex differences exist in the incidence of AF, with women having a lower incidence of AF compared to men [2, 3]. However, women with AF experience greater symptomatology, functional impairment and worse quality of life compared to their male counterparts [4, 5]. Women with AF also have a higher risk of stroke (more evident at age over 65 years), and all-cause and cardiovascular mortality as compared to men [6–8]. Despite their higher symptom burden and stroke risk, women with AF are less likely to (1) be seen by a cardiologist, (2) receive rhythm control treatment, including cardioversion and AF ablation, and (3) be prescribed appropriate anticoagulation [5, 9–12]. However, little is known about potential sex differences in the use of specific treatment strategies and adverse outcomes among the elderly (aged 75 years and older). This is particularly relevant given the higher risk of stroke and other complications among this group and the pervasive nature of health inequities between men and women, even at older ages [13, 14].

This study aims to explore the association of biological sex with treatment of AF and outcomes among men and women over 75 years with claims for AF in the IBM MarketScan database. Our primary aim is to estimate potential sex differences in the initiation of AF treatments, specifically anticoagulant therapy and rate versus rhythm control strategy, among newly diagnosed patients with AF. Secondarily, we aim to evaluate the association of sex with cardiovascular (stroke and heart failure hospitalization) and major bleeding outcomes as well as the interaction between sex and treatment strategy with regard to these outcomes. We hypothesized that women were less likely to receive anticoagulation than men and, among those receiving anticoagulation, more women would initiate anticoagulation on warfarin [vs. direct oral anticoagulants (DOACs)] as compared to men. We also hypothesized sex interactions in the associations of anticoagulant use and rate control treatment with outcomes, with women on these therapies experiencing greater adverse health outcomes as compared to men with a similar risk factor profile.

## Methods

### Study population

We used data from the IBM MarketScan Medicare Supplemental and Coordination of Benefits (Medicare) Database (IBM Watson Health) from January 1, 2007 through October 1, 2015. The MarketScan Medicare database contains individual level healthcare claims and enrollment data from individuals and their dependents with Medicare supplemental plans within the United States [15]. The database contains claims for inpatient and outpatient services, as well as outpatient pharmacy claims.

**Fig. 1:**
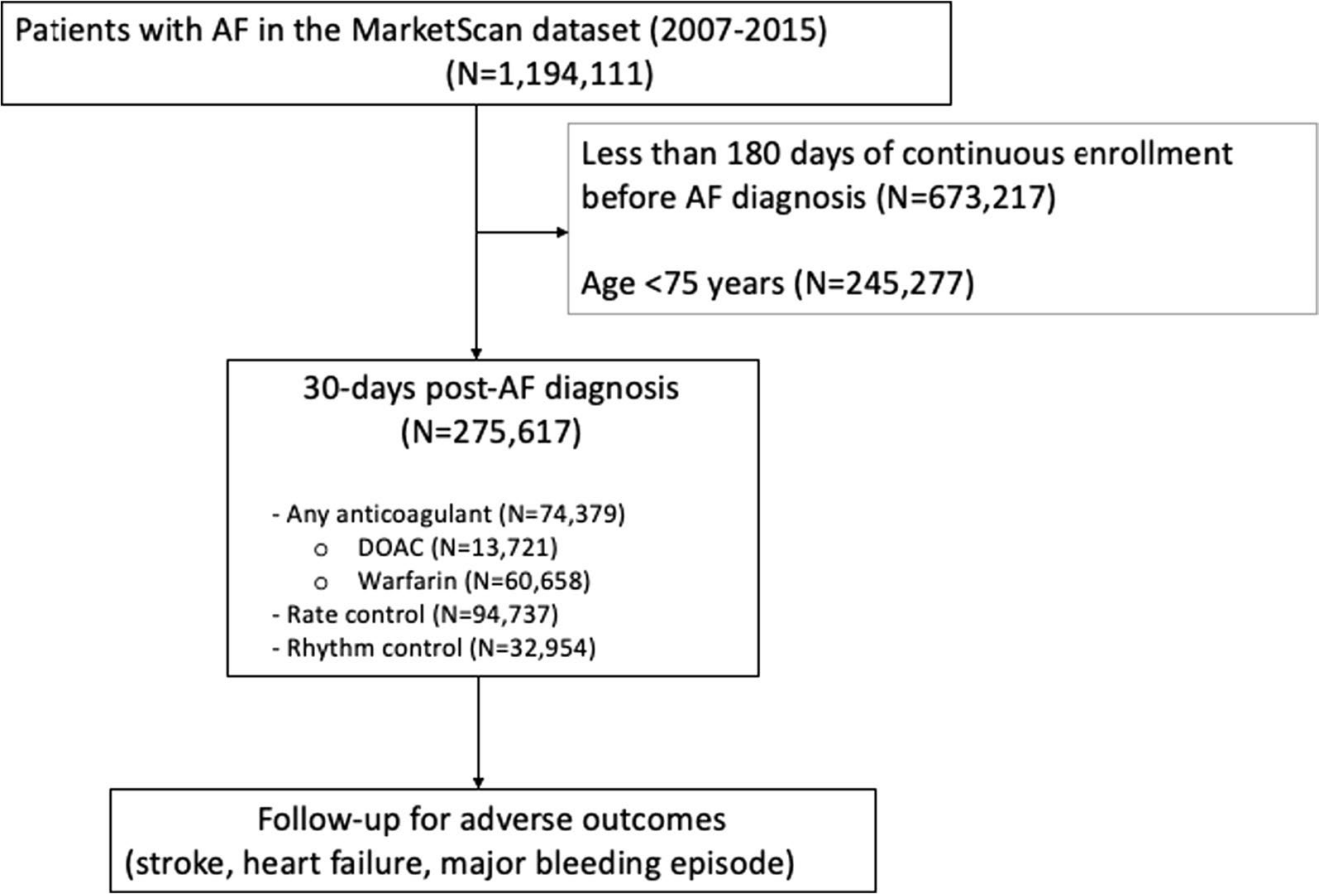
Flow chart of participant selection into the study sample. *AF* atrial fibrillation, *DOAC* direct oral anticoagulants

In the present analysis, the study population consisted of participants enrolled in the MarketScan database at some point between January 1, 2007 and October 1, 2015, who were continuously enrolled for 180 days prior to receiving a diagnosis of non-valvular AF. Valvular AF, defined in this analysis as AF with a history of mitral stenosis (ICD-9-CM 394.0) or mitral valve disorder (ICD-9-CM 424.0), is a contraindication for DOACs and, in general, has a different management and has been excluded from this analysis. AF was diagnosed using International Classification of Diseases, Ninth Revision, Clinical Modification (ICD-9-CM) codes, 427.31 and 427.32 for one inpatient or two outpatient claims between 7 days and a year apart, in any position [16]. The final study sample consisted of 275,617 men and women (Fig. 1).

### Treatment strategy

Oral anticoagulant use (warfarin and DOACs) within 30 days after the initial diagnosis of AF was assessed from outpatient pharmacy claims. Oral anticoagulant use claims were included independent of the dose prescribed, assuming patients were prescribed the correct dosage, to create a binary variable for anticoagulant use. Previously, it has been shown that the validity of warfarin claims in administrative databases has a high positive predictive value of 99% [17].

Rate and rhythm control treatment strategies within 30 days after the initial diagnosis of AF were ascertained from pharmacy, inpatient and outpatient claims. Rate control was defined as receipt of any of the following treatments: beta-blockers, non-dihydropyridine calcium channel blockers, digoxin or atrioventricular node ablation procedure (CPT code 93650) without a pacemaker. Rhythm control was defined by the presence of any of the following: receipt of antiarrhythmic medication, catheter ablation procedure (CPT code 93651 (before 2013), 93656 or 93657 (after January 2013) or ICD-9-CM procedure code 37.34 in the absence of codes for pacemaker or implantable cardioverter defibrillator implementation, or AV node ablation) and cardioversion (ICD-9-CM code 99.61, 99.62, or CPT code 92960, 92961). Participants who received both rate and rhythm control treatment were classified under rhythm control.

### Outcome events

The outcome variables were initial hospitalization for a major bleeding episode, heart failure and ischemic stroke, after a diagnosis of AF. These outcomes were identified from the primary diagnosis of hospitalizations using ICD-9-CM codes and applying validated algorithms [18–20]. Specific codes used to define each outcome are provided in Additional file 1: Supplementary Table 1.

### Sex and other covariates

Sex was determined at entry into the MarketScan database by patient-level demographic information and defined as male and female. Co-morbidities and medication use determined a priori to be risk factors for these outcomes were identified using ICD-9-CM codes from inpatient, outpatient, and pharmacy claims prior to the time of diagnosis of AF, using previously developed algorithms [21]: age at diagnosis, excessive alcohol consumption, congestive heart failure, hypertension, diabetes mellitus, hyperlipidemia, stroke, coronary artery disease, myocardial infarction, chronic kidney disease, gastrointestinal bleed, disorders of the liver, cancer, chronic obstructive pulmonary disease, dementia, depression, intracranial and other bleeds. Claims data were also used to obtain information on prevalent medication at the time of AF diagnosis: lipid lowering medication, beta-blockers, calcium channel blockers, angiotensin converting enzyme inhibitors, diuretics, gastrointestinal medication, other cardiac medication, potassium supplements, cardiac glycosides, antidiabetics, antiplatelets, thiazide diuretics, antiarrhythmics, insulin, sulphonylureas, other diuretics, statins, digoxin and angiotensin receptor blockers.

### Statistical analysis

Poisson regression with robust error variance was used to estimate the relative risk for the association between sex and initial treatment strategy within 30-days post diagnosis of AF. Cox proportional hazards models were used to estimate the association of treatment, post-AF diagnosis with time to an initial hospitalization of each of the outcomes (stroke, major bleeding and heart failure). Each outcome was fit in a separate Cox model. Treatment was determined by what participants received in the 30-day period after a diagnosis of AF, with follow-up time to the outcome beginning at that 30-day mark, thus excluding those who did not start treatment or who died during this time frame. In all models, we adjusted for age initially and then for all the other risk factors and medications listed above. We tested for both multiplicative and additive (through assessment of the interaction contrast ratio) interaction of treatment choice with sex and also fit sex-stratified Cox proportional hazard models for each outcome. All analyses were run on SAS version 9.4 (SAS Inc, Cary, NC).

## Results

### Participant characteristics

The characteristics of the study population at the time of AF diagnosis (N = 275,617), stratified by sex are represented in Table 1. The average age (SD) of the population was 83.2 (5.4) years. Women were slightly older than men (83.8 vs. 82.5, respectively). Women were more likely to have hypertension, dementia or depression. They were also more likely to be prescribed calcium channel blockers and digoxin. Men were more likely to have a diagnosis of diabetes mellitus, coronary artery disease, chronic kidney disease and hyperlipidemia. They were also more likely to be prescribed anticoagulants, anti-platelet medication, anti-arrhythmic drugs, angiotensin converting enzyme inhibitors and statins.

**Table 1:** Participant characteristics at time of atrial fibrillation diagnosis, MarketScan database, 2007–2015

	Overall (N = 275,617)	Men (N = 135,850)	Women (N = 139,767)
Age, years	83.2 (5.4)	82.5 (5.2)	83.8 (5.6)
Prior medical history			
Alcohol use	2520 (0.9)	1782 (1.3)	738 (0.5)
Chronic kidney disease	68,521 (24.9)	37,522 (27.6)	30,999 (22.2)
Chronic obstructive pulmonary disease	72,016 (26.1)	36,890 (27.2)	35,126 (25.1)
Coronary artery disease	128,965 (46.8)	74,934 (55.2)	54,031 (38.7)
Diabetes mellitus	87,876 (31.9)	47,727 (35.1)	40,149 (28.7)
Dementia	38,528 (14.0)	16,728 (12.3)	21,800 (15.6)
Depression	23,461 (8.5)	8774 (6.5)	14,687 (10.5)
Gastrointestinal bleed	29,117 (10.6)	14,496 (10.7)	14,621 (10.5)
Heart failure	89,013 (32.2)	44,330 (32.6)	44,683 (32.0)
Hyperlipidemia	130,423 (47.3)	67,345 (49.6)	63,078 (45.1)
Hypertension	210,052 (76.2)	100,749 (74.2)	109,303 (78.2)
Intracranial bleed	5527 (2.0)	2775 (2.0)	2752 (2.0)
Liver disease	10,918 (4.0)	5542 (4.1)	5376 (3.9)
Myocardial infarction	30,809 (11.2)	17,410 (12.8)	13,399 (9.6)
Other bleeds	34,100 (12.4)	21,094 (15.5)	13,006 (9.3)
Peripheral arterial disease	54,238 (19.7)	27,967 (20.6)	26,271 (18.8)
Stroke	81,717 (29.7)	40,556 (29.9)	41,161 (29.5)
Medication use			
Angiotensin converting enzyme inhibitors	97,532 (35.4)	51,987 (38.3)	45,545 (32.6)
Angiotensin receptor blockers	63,464 (23.0)	27,054 (19.9)	36,410 (26.1)
Anti-arrhythmic medication	31,546 (11.5)	16,644 (12.3)	14,902 (10.7)
Anticoagulant	111,537 (40.5)	58,314 (42.9)	53,223 (38.1)
Anti-platelet medication	45,884 (16.7)	25,382 (18.7)	20,502 (14.7)
Beta-blockers	164,968 (59.9)	80,043 (59.0)	84,925 (60.8)
Calcium channel blockers	104,583 (40.0)	45,452 (33.5)	59,131 (42.3)
Digoxin	43,501 (15.8)	20,388 (15.0)	23,113 (16.5)
Diuretics	94,469 (34.3)	45,436 (33.5)	49,033 (35.1)
Gastrointestinal drugs	83,380 (30.3)	38,682 (28.5)	44,678 (32.0)
Glucose-lowering medication	37,508 (13.6)	21,119 (15.6)	16,389 (11.7)
Insulin	17,370 (6.3)	9518 (7.0)	7852 (5.6)
Lipid lowering medication	32,841 (11.9)	18,000 (13.3)	14,841 (10.6)
Other diuretics	31,493 (11.4)	13,907 (10.2)	17,586 (12.6)
Potassium supplements	61,331 (22.3)	26,417 (19.5)	34,914 (25.0)
Statins	145,269 (52.7)	77,585 (57.1)	67,684 (48.4)
Sulphonylureas	28,226 (10.2)	16,319 (12.0)	11,907 (8.5)

Age is presented as mean (standard deviation), and all other characteristics are presented as N (%)

### Association of sex with choice of treatment strategy

Overall 26.3% women received an anticoagulant during the 30 days after diagnosis, as did 27.7% of men. Women were slightly less likely to receive an anticoagulant (vs. none) within 30-days post diagnosis of AF after adjusting for age, cardiovascular risk factors and relevant medication [relative risk (RR) 0.94, 95% confidence interval (CI) 0.93, 0.96] (Table 2). Among OAC users, 18.7% of women and 18.2% of men used a DOAC. After adjustment, among those receiving oral anticoagulation, women had a slightly higher probability of receiving DOACs (vs. warfarin) (RR 1.05, 95% CI 1.01, 1.08). Rhythm control (vs. rate control) treatment was less common in women than in men after adjustment for age (RR 0.85, 95% CI 0.83, 0.86) and other risk factors (RR 0.94, 95% CI 0.93, 0.96).

**Table 2:** Association of biological sex with treatment choice within 30-days after a diagnosis of atrial fibrillation among elderly participants in the MarketScan database (2007–2015)

Treatment 30-day after diagnosis	Men	Women
Any anticoagulant (vs. none)		
Events/N	37,574/135,850	36,805/139,767
%	27.7	26.3

	RR (95% CI)	
Model 1^†^	1 (ref.)	1.00 (0.98, 1.01)
Model 2^††^	1 (ref.)	**0.94 (0.93, 0.96)**
DOAC (vs. warfarin)		
Events/N	6821/37,574	6900/36,805
%	18.2	18.7

	RR (95% CI)	
Model 1^†^	1 (ref.)	**1.04 (1.01, 1.07)**
Model 2^††^	1 (ref.)	**1.05 (1.01, 1.08)**
Rhythm control (vs. rate)		
Events/N	17,232/60,053	15,722/67,638
%	28.7	23.2

	RR (95% CI)	
Model 1^†^	1 (ref.)	**0.85 (0.83, 0.86)**
Model 2^††^	1 (ref.)	**0.94 (0.93, 0.96)**

*RR* risk ratio, *CI* confidence interval, *DOAC* direct oral anticoagulants

Bolded results are statistically significant at *p* value of 0.05

^†^Model 1 adjusts for age

^††^Model 2 adjusts for age plus heart failure, hypertension, diabetes mellitus, stroke, myocardial infarction, peripheral arterial disease, chronic kidney disease, gastrointestinal bleed, liver disease, hyperlipidemia, chronic obstructive pulmonary disease, depression, dementia, intracranial bleeding, other forms of bleeding, alcohol abuse, use of lipid lowering medication, beta blockers, calcium channel blockers, angiotensin receptor blocker, angiotensin converting enzyme inhibitor, diuretics, gastrointestinal drugs, cardiac drugs, potassium supplements, anti-diabetics, anti-platelet drugs, thiazide diuretics, anti-arrhythmics, insulin, sulphonylureas, other diuretics, statins, digoxin and oral anticoagulant use (in models assessing initiation of Rhythm v. Rate control)

### Sex, anticoagulation and adverse outcomes

Among male AF patients, those receiving any anticoagulant (as compared to those not on an anticoagulant) had a higher hazard of incident heart failure [hazard ratio (HR) 1.17, 95% CI 1.11, 1.22] and major bleeding (HR 1.24, 95% CI 1.18, 1.30) and a lower hazard of stroke (HR 0.84, 95% CI 0.78, 0.90), after adjusting for age (Table 3). Similar results were seen after adjusting for other cardiovascular risk factors. Women on any anticoagulant (vs. no anticoagulant) also had a higher hazard of heart failure [1.26 (1.20, 1.31)] and major bleeding [1.29 (1.23, 1.36)] and a lower hazard for stroke [0.91 (0.86, 0.97)], after adjusting for age and similarly after adjustment for other risk factors. We identified a significant multiplicative interaction between sex and anticoagulant initiation in relationship to heart failure hospitalization, with the risk of heart failure associated with oral anticoagulation being stronger in women than men. On the additive scale, we found positive additive interaction for sex and anticoagulation in relation to each of the three adverse outcomes (interaction contrast ratio (ICR) of 0.076 for heart failure, 0.068 for stroke and 0.056 for major bleeding episodes) (Table 3).

**Table 3:** Association of anticoagulant use (vs. none) after atrial fibrillation diagnosis with cardiovascular events, by sex, among elderly participants in the MarketScan database (2007–2015)

Outcome	Men				Women				*p* value for multiplicative interaction	ICR
	Model 1^†^		Model 2^††^		Model 1^†^		Model 2^††^			
	Anticoagulant	Anticoagulant	Anticoagulant	Anticoagulant	Anticoagulant	Anticoagulant	Anticoagulant	Anticoagulant		
	**−**	**+**	**−**	**+**	**−**	**+**	**−**	**+**		
Heart failure										
Events/N	4020/64,656	5154/62,653	4020/64,656	5154/62,653	4093/72,956	4461/57,975	4093/72,956	4461/57,975	0.003	0.076
HR (95% CI)	1 (ref.)	**1.17 (1.11, 1.22**)	1 (ref.)	**1.05 (1.01, 1.10)**	1 (ref.)	**1.26 (1.20, 1.31)**	1 (ref.)	**1.13 (1.08, 1.18)**		
Stroke										
Events/N	1526/64,884	1461/62,898	1526/64,884	1461/62,898	2341/73,149	1906/58,186	2341/73,149	1906/58,186	0.11	0.068
HR (95% CI)	1 (ref.)	**0.84 (0.78, 0.90)**	1 (ref.)	**0.83 (0.77, 0.90)**	1 (ref.)	**0.91 (0.86, 0.97)**	1 (ref.)	**0.91 (0.85, 0.97)**		
Major bleeding										
Events/N	2154/63,733	4100/63,878	2154/63,733	4100/63,878	2279/70,318	3855/60,944	2279/70,318	3855/60,944	0.13	0.056
HR (95% CI)	1 (ref.)	**1.24 (1.18, 1.30)**	1 (ref.)	**1.28 (1.22, 1.35)**	1 (ref.)	**1.29 (1.23, 1.36)**	1 (ref.)	**1.35 (1.28, 1.42)**		

*HR* hazard ratio, *CI* confidence interval, *ICR* interaction contrast ratio

Bolded results are statistically significant at *p* value of 0.05

^†^Model 1 adjusts for age

^††^Model 2 adjusts for age plus heart failure, hypertension, diabetes mellitus, stroke, myocardial infarction, peripheral arterial disease, chronic kidney disease, gastrointestinal bleed, liver disease, hyperlipidemia, chronic obstructive pulmonary disease, depression, dementia, intracranial bleeding, other forms of bleeding, alcohol abuse, use of lipid lowering medication, beta blockers, calcium channel blockers, angiotensin receptor blocker, angiotensin converting enzyme inhibitor, diuretics, gastrointestinal drugs, cardiac drugs, potassium supplements, anti-diabetics, anti-platelet drugs, thiazide diuretics, anti-arrhythmics, insulin, sulphonylureas, other diuretics, statins and digoxin

We evaluated the association of the use of DOACs (compared to warfarin) with outcomes (Table 4). Participants on DOAC had a lower hazard of heart failure (HR 0.81, 95% CI 0.73, 0.91 in men and HR 0.82, 95% CI 0.73, 0.92 in women) after adjusting for age. Associations were attenuated after adjusting for other cardiovascular risk factors. Analyses did not provide evidence of differences in stroke or bleeding risk by type of oral anticoagulant between men and women on the multiplicative scale. However, on the additive scale, we found evidence of a positive additive interaction for sex and DOAC usage in relation to bleeding (ICR = 0.063), stroke (ICR = 0.119) and heart failure hospitalization (ICR = 0.02).

**Table 4:** Association of Direct oral anticoagulant use (vs. warfarin) after atrial fibrillation diagnosis with cardiovascular events, by sex, among elderly participants in the MarketScan database (2007–2015)

Outcome	Men				Women				*p* value for multiplicative interaction	ICR
	Model 1^†^		Model 2^††^		Model 1^†^		Model 2^††^			
	Warfarin	DOAC	Warfarin	DOAC	Warfarin	DOAC	Warfarin	DOAC		
Heart failure										
Events/N	4799/54,192	355/8461	4799/54,192	355/8461	4149/49,845	312/8130	4149/49,845	312/8130	0.89	0.02
HR (95% CI)	1 (ref.)	**0.81 (0.73, 0.91)**	1 (ref.)	0.94 (0.84, 1.05)	1 (ref.)	**0.82 (0.73, 0.92)**	1 (ref.)	0.93 (0.83, 1.04)		
Stroke										
Events/N	1328/54,411	93/8487	1328/54,411	93/8487	1766/50,031	140/8155	1766/50,031	140/8155	0.3	0.119
HR (95% CI)	1 (ref.)	0.85 (0.69, 1.05)	1 (ref.)	0.89 (0.72, 1.10)	1 (ref.)	1.00 (0.84, 1.19)	1 (ref.)	1.01 (0.86, 1.22)		
Major bleeding										
Events/N	3390/54,315	286/8481	3390/54,315	286/8481	3066/49,981	273/8144	3066/49,981	273/8144	0.56	0.063
HR (95% CI)	1 (ref.)	0.99 (0.88, 1.12)	1 (ref.)	1.01 (0.89, 1.14)	1 (ref.)	0.99 (0.87, 1.12)	1 (ref.)	1.03 (0.90, 1.16)		

*HR* hazard ratio, *CI* confidence interval, *ICR* interaction contrast ratio

Bolded results are statistically significant at *p* value of 0.05

^†^Model 1 adjusts for age

^††^Model 2 adjusts for age plus heart failure, hypertension, diabetes mellitus, stroke, myocardial infarction, peripheral arterial disease, chronic kidney disease, gastrointestinal bleed, liver disease, hyperlipidemia, chronic obstructive pulmonary disease, depression, dementia, intracranial bleeding, other forms of bleeding, alcohol abuse, use of lipid lowering medication, beta blockers, calcium channel blockers, angiotensin receptor blocker, angiotensin converting enzyme inhibitor, diuretics, gastrointestinal drugs, cardiac drugs, potassium supplements, anti-diabetics, anti-platelet drugs, thiazide diuretics, anti-arrhythmics, insulin, sulphonylureas, other diuretics, statins and digoxin

### Sex, rhythm versus rate control therapy, and adverse outcomes

We assessed the association of rhythm control strategy (compared to rate control) with adverse outcomes (Table 5). In men, rhythm control was associated with a greater risk for heart failure (HR 1.20, 95% CI 1.14, 1.25) and a lower risk for ischemic stroke (HR 0.73, 95% CI 0.67, 0.80). There was no evidence of meaningful association of rhythm (vs. rate) control therapy with the risk of major bleeding (HR 1.04, 95% CI 0.98, 1.10). These results were seen in the age-adjusted model. Among women, we saw similar results with slightly larger point estimates. Women on any rhythm control had a higher hazard of heart failure (HR 1.34, 95% CI 1.28, 1.40) and a lower hazard of ischemic stroke (HR 0.81, 95% CI 0.75, 0.87) than those on rate control therapy. There was no association between rhythm (vs. rate control) and an episode of major bleeding (HR 1.01, 95% CI 0.95, 1.07), after adjusting for age. There was a significant multiplicative interaction between sex and treatment, with the association of rhythm (vs. rate) control therapy with heart failure risk being stronger in women than men. Also, there was a positive additive interaction between sex and use of rhythm (vs. rate) control treatment for the risk for stroke and heart failure. There was a negative additive interaction for sex and rhythm control (v. rate) and bleeding, but the magnitude of this interaction was small (− 0.03).

**Table 5:** Association of rhythm (vs. rate) control after atrial fibrillation diagnosis with cardiovascular events, by sex, among elderly participants in the MarketScan database (2007–2015)

Outcome	Men				Women				*p* value for multiplicative interaction	ICR
	Model 1^†^		Model 2^††^		Model 1^†^		Model 2^††^			
	Rate control	Rhythm control	Rate control	Rhythm control	Rate control	Rhythm control	Rate control	Rhythm control		
Heart failure										
Events/N	5290/65,843	3052/31,047	5290/65,843	3052/31,047	5121/75,251	2761/28,753	5121/75,251	2761/28,753	< 0.0001	0.152
HR (95% CI)	1 (ref.)	**1.20 (1.14, 1.25)**	1 (ref.)	**1.21 (1.15, 1.28)**	1 (ref.)	**1.34 (1.28, 1.40)**	1 (ref.)	**1.41 (1.34, 1.49)**		
Stroke										
Events/N	1838/66,102	682/31,209	1838/66,102	682/31,209	2829/75,465	961/28,899	2829/75,465	961/28,899	0.08	0.071
HR (95% CI)	1 (ref.)	**0.73 (0.67, 0.80)**	1 (ref.)	**0.79 (0.72, 0.87)**	1 (ref.)	**0.81 (0.75, 0.87)**	1 (ref.)	**0.85 (0.78, 0.92)**		
Major bleeding										
Events/N	3529/66,041	1876/31,143	3529/66,041	1876/31,143	3756/75,413	1664/28,866	3756/75,413	1664/28,866	0.99	− 0.03
HR (95% CI)	1 (ref.)	1.04 (0.98, 1.10)	1 (ref.)	**1.07 (1.01, 1.14)**	1 (ref.)	1.01 (0.95, 1.07)	1 (ref.)	1.02 (0.96, 1.09)		

*HR* hazard ratio, *CI* confidence interval, *ICR* interaction contrast ratio

Bolded results are statistically significant at *p* value of 0.05

^†^Model 1 adjusts for age

^††^Model 2 adjusts for age plus heart failure, hypertension, diabetes mellitus, stroke, myocardial infarction, peripheral arterial disease, chronic kidney disease, gastrointestinal bleed, liver disease, hyperlipidemia, chronic obstructive pulmonary disease, depression, dementia, intracranial bleeding, other forms of bleeding, alcohol abuse, use of lipid lowering medication, beta blockers, calcium channel blockers, angiotensin receptor blocker, angiotensin converting enzyme inhibitor, diuretics, gastrointestinal drugs, cardiac drugs, potassium supplements, anti-diabetics, anti-platelet drugs, thiazide diuretics, anti-arrhythmics, insulin, sulphonylureas, other diuretics, statins, digoxin and oral anticoagulant use

## Discussion

This study found, in a healthcare claims database, treatment of AF and the association between treatment choice and outcomes among elderly patients with AF differed by sex. Elderly patients with AF represent a unique clinical situation with a high prevalence of polypharmacy and multiple comorbidities that may alter or limit the use of specific therapies. Characterizing potential sex disparities in treatment and outcomes of AF can identify gaps in care and could be key to improve outcomes among this patient group. Specifically, we found that elderly women are less likely than men to be prescribed an anticoagulant or receive rhythm control treatment after a diagnosis of AF. Participants with a diagnosis of AF receiving an anticoagulant were at a higher risk for heart failure and a major bleeding episode but at a lower risk for an ischemic stroke. Within anticoagulants, DOACs were associated with lower risk of heart failure (vs. warfarin), but not of stroke or bleeding. Rhythm control (vs. rate control) was associated with an increased risk for heart failure and a lower risk for ischemic stroke. The associations of anticoagulant therapy and rhythm control with heart failure was significantly stronger in women compared to men.

### Sex differences in AF treatment

Our study found that women aged ≥ 75 years were less likely to be prescribed an oral anticoagulant compared to men. This finding is consistent with some previous studies [9, 22]. In the Canadian Registry of Atrial Fibrillation, men with AF, aged ≥ 75 years and the presence of > 1 stroke risk factor were significantly more likely to receive warfarin as compared to women with a similar risk profile (44.9% vs. 24.5%) [23]. Similarly, in the PINNACLE National Cardiovascular Data Registry, women were less likely to use an OAC than men, overall and at all levels of the CHA_2_DS_2_-VASc score [24]. The PINNACLE study also found that DOAC use was increasing among women in the study [24].

Considering differences in use of OACs across a broader age-range, contrary to our findings, data from more recent observational cohort, registry and administrative studies appear to suggest that there is less evidence in support of the existence of sex disparities in use of OAC’s for atrial fibrillation [4, 25–28]. In the Euro Observational Research Programme on Atrial Fibrillation (EORP-AF) Pilot survey, among men and women aged > 65 years, a greater number of women received oral anticoagulation after an AF diagnosis (women vs. men, 95.3 vs. 76.2%) [25]. In the Outcomes Registry for Better Informed Treatment of Atrial Fibrillation (ORBIT-AF) study, there were similar rates of anticoagulation among men and women [4]. The change in observed sex differences in anticoagulation could be related to the inclusion of the CHA_2_DS_2_-VASc score in guideline-based management for AF [29]. This score includes female sex as a risk factor for thromboembolic events and may have led to more women with AF receiving an OAC. Adoption of these guidelines could also have led to a decrease in the observed differences in OAC use between men and women.

As in our analysis, other studies have found that women are less likely than men to receive rhythm control therapy as compared to rate control [4, 22, 25–28, 30]. Among women receiving anti-arrhythmic therapy, they were also less likely to receive AV-node ablation or cardioversion [22, 31]. This has been attributed to the perception of prescribers that biological differences between men and women could lead to worse health outcomes in women. Women require higher doses of AV nodal agents to address the greater noradrenergic reponse seen in them [32, 33]. Women also tend to have a longer baseline QT_c_ interval (secondary to sex hormone levels) than men, which makes them more susceptible to developing torsades de pointes on certain class III and class Ia anti-arrhythmics [34, 35].

### Oral anticoagulation and adverse outcomes

In this study we found that men and women on any anticoagulant were at a slightly higher risk for heart failure, particularly among women, and a major bleeding episode and at a lower risk for ischemic stroke. The observed increased risk of heart failure among those receiving anticoagulation, particularly women, is unexpected, and could be explained by residual confounding or this could be a chance finding.

### DOACs versus warfarin and outcomes

When comparing the effect of DOAC (vs. warfarin) on outcomes, we found DOACs were associated with lower risk of heart failure in both men and women. A recent study found that DOACs, compared to warfarin, were associated with a lower risk for intracranial hemorrhage and all-cause mortality in women with no difference in the risk for stroke, systemic embolism and gastrointestinal bleeds [36]. Similarly, in a registry-based analysis from Japan on patients with AF on DOAC’s, despite women in the study having a higher bleeding risk, there was no meaningful difference in bleeding episodes but they did experience a higher risk for thromboembolic events [37]. Among participants on DOACs, other studies have found lower risk for a major bleeding episode among women [38, 39] while men experienced lower risk of stroke and systemic embolism [39]. A meta-analysis of randomized controlled trials comparing DOACs to warfarin confirmed these findings [40]. In this study we did not see a lower risk of major bleeding when on DOACs, which may be related to residual confounding, or specific to this older age population.

### Rhythm versus rate control and adverse outcomes

This study found that rhythm control (vs. rate control) strategies were associated with a higher risk for heart failure, a lower risk for stroke and had no effect on major bleeding episodes in both men and women. The association of rhythm control with heart failure risk was slightly stronger in women than men. Some of our results concur with observations from landmark clinical trials. The AFFIRM trial, conducted in an elderly population (mean age 75 years), found no difference in the risk of stroke or major bleeding with rate control (vs. rhythm control) [41]. The ORBIT-AF registry study, also conducted in the elderly (mean age 74 years) found in the unadjusted analyses that rhythm control was associated with a lower risk for stroke, major bleeding and CV mortality, these results did not stay the same when adjusting for risk factors [42]. A post-hoc analysis of the AFFIRM study [43], RECORD-AF [44] and observational data [45] found that long-term rhythm control lowered mortality and hospitalization. The increased risk in heart failure in our study on rhythm control may be related to the age of our participants and the presence of multiple co-morbidities at baseline predisposing them to the adverse effects of anti-arrhythmic drugs and other rhythm control approaches. Among women, a higher risk for HF in AF may be related to treatment strategy which is in turn related to healthcare provider (generalist vs. specialist) and perhaps a longer lifespan which could allow for a longer time to development of HF in AF. We also noted a lower risk for stroke with rhythm control in the MarketScan population, perhaps pointing to adequate anticoagulation among those receiving rhythm control or residual confounding.

### Strengths and limitations

Our results should be interpreted considering the study limitations. Our primary concern is uncontrolled confounding: we did not have detailed clinical data, such as symptom burden, type of AF (paroxysmal, persistent, permanent), heart rate, or presence of hemodynamic instability at time of diagnosis, that could help inform treatment decisions and provide information on prescribing patterns. We used diagnostic codes for all variables in the analysis and potential for misclassification exists. Where possible, however, we used validated algorithms to minimize this misclassification.

Our study also has several strengths. The claims data we used is geographically diverse, containing data from Medicare enrollees (with supplemental insurance) from across the United States. We had access to risk factor information, allowing for adjustment and the study of effect modification in our analysis. Due to the comprehensive nature of the database, we had a large study sample size of participants aged 75 years and older. Database enrollment and censoring due to disenrollment are unlikely to be related to exposures and endpoints, limiting the risk of selection bias.

In conclusion, this study identified sex differences in treatment of elderly patients with AF as well as response to such treatments. We found that elderly women were less likely to receive any anticoagulation or rhythm control as compared to men. While both men and women on anticoagulants and/or rhythm control had an increased risk for heart failure, women had a modestly higher risk. This study adds to the body of evidence highlighting the importance of understanding sex differences in treatment and outcomes in an elderly population with atrial fibrillation and with a high prevalence of co-morbidities and polypharmacy. Future studies will need to delve into determinants of prescribing patterns and underlying mechanisms to understand the basis of these sex differences and develop strategies to resolve them.

## Supplementary Information

**Additional file 1.** Supplementary Table 1.

## Data Availability

Data used in the current analysis cannot be deposited in a public repository due to licensing and privacy restrictions. Access to this data was approved by the Emory University Institutional Review Board. Individuals interested in obtaining the MarketScan data used in the current analysis should contact IBM Watson Health using the contact form provided at https://www.ibm.com/products/marketscan-research-databases/databases.

## Abbreviations

AF: Atrial fibrillation
CVD: Cardiovascular disease
DOAC’s: Direct oral anticoagulants
AV: Atrioventricular
