# Supplementary material for: Sex differences in treatment strategy and adverse outcomes among patients 75 and older with atrial fibrillation in the MarketScan database
Source: BMC Cardiovasc Disord. 2021 Dec 16;21:598. doi: 10.1186/s12872-021-02419-2 (PMC8679994; doi:10.1186/s12872-021-02419-2)
Supplement: Supplementary file 1 — Additional file 1. Supplementary Table 1. [file 12872_2021_2419_MOESM1_ESM.docx]

**Supplementary Table 1:** ICD-9-CM codes used to define comorbidities

| **Co-morbidity** | **ICD-9 CM codes (3 or 4 or 5 digit)** |
| --- | --- |
| Hypertension | 401, 402, 403, 404, 405, 4372, 36211 |
| Congestive heart failure | 39891, 40201, 40211, 40291, 40401, 40403, 40411, 40413, 40491, 40493, 428 |
| Coronary heart disease | 410, 411, 412, 413, 4140, 4142, 4143, 4148, 4149, 41412, V4581, V45826 |
| Hyperlipidemia | 2720, 2721, 2722, 2723, 2724 |
| Stroke | 430, 431, 432, 433, 434, 434, 435, 436, 437, 3466, 99702 |
| Chronic Kidney Disease | 23691, 24940, 24941, 27410, 28311, 40301, 40311, 40391, 40402, 40403, 40412, 40413, 40492, 40493, 75312, 75313, 75314, 75315, 75316, 75317, 75319, V4511, V4512, V5631, V5632, 1890, 1899, 2230, 2504, 2714, 4401, 4421, 5724, 7532, 7925, 7944, 0160, 0954, V420, V451, V560, V561, V562, V568, 580, 581, 582, 583, 584, 585, 586, 587, 588, 591 |
| Chronic pulmonary disease | 490, 491, 492, 494, 496 |
| Dementia | 290, 294, 797, 2930, 2931, 3100, 3102, 3108, 3109, 3310, 3311, 3312, 3317, 31081, 31089, 33111, 33119, 33182 |
| Depression | 311, 2962, 2963, 2966, 2980, 3004, 3091, 29651, 29652, 29653, 29654, 29655, 29656, 29689 |
| Diabetes | 249, 250, 3572, 7902, 7902, 7915, 7916, 36201, 36202, 36641, V4585, V5391, V6546 |
| Peripheral artery disease | 4400, 4402, 4409, 4439 |
| Liver disease | 07022, 07023, 07032, 07033, 07044, 07054, 0706, 0709, 4560, 4561, 4562, 5722, 5723, 5724, 5725, 5726, 5727, 5728, 5733, 5734, 5738, 5739, V427, 570, 571 |
| GI bleeding | 45620, 53082, 53501, 53511, 53521, 53531, 53541, 53551, 53561, 53783, 56202, 56203, 56212, 56213, 56881, 56985, 4552, 4555, 4558, 4560, 5307, 5310, 5312, 5314, 5316, 5320, 5322, 5324, 5326, 5330, 5332, 5334, 5336, 5340, 5342, 5344, 5346, 5693, 5780, 5781, 5789 |
| Prior cerebral bleeding | 430, 431, 432, 852 |
| Other bleeding | 56881, 59381, 4230, 4590, 5997, 6238, 6266, 7191, 7847, 7848, 7863 |
| Myocardial Infarction | 410, 412 |
| Alcoholism | 291, 303, 3050, 3575, 4255, 5353, 5710, 5711, 5712, 5713, 9800, 76071 |
